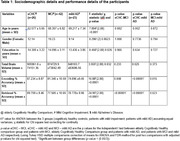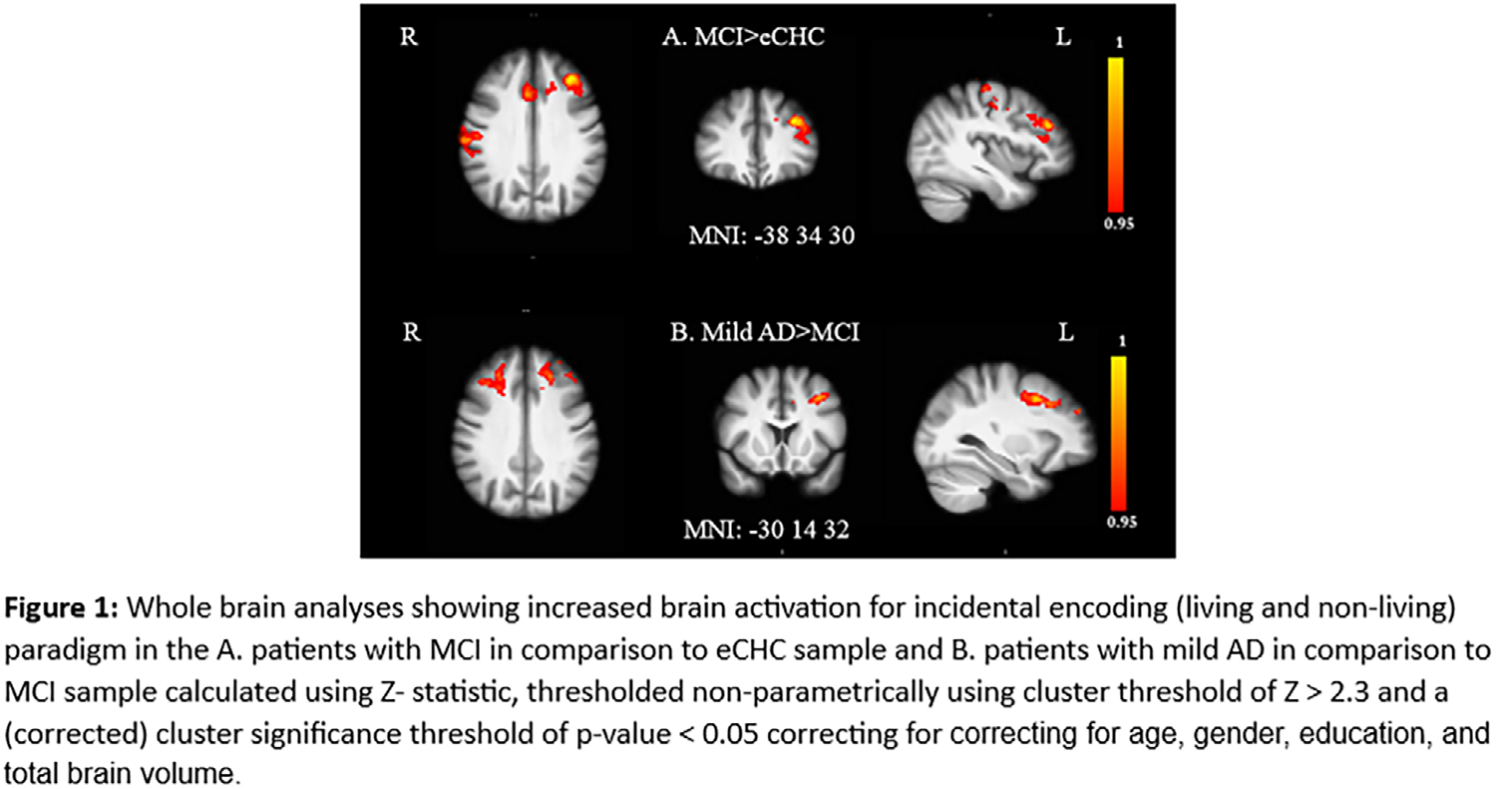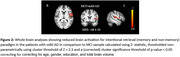# A novel fMRI memory paradigm designed for brain imaging studies in early Alzheimer’s Disease

**DOI:** 10.1002/alz.094041

**Published:** 2025-01-09

**Authors:** Himanshu Joshi, PT Sivakumar, Ganesan Venkatasubramanian, John P John, Paul M. Thompson

**Affiliations:** ^1^ Multimodal Brain Image Analysis Laboratory, National Institute of Mental Health and Neurosciences (NIMHANS), Bengaluru, Karnataka India; ^2^ Departmet of Psychiatry, National Institute of Mental Health and Neurosciences (NIMHANS), Bengaluru, Karnataka India; ^3^ Translational Psychiatry Laboratory, National Institute of Mental Health and Neurosciences (NIMHANS), Bengaluru, Karnataka India; ^4^ Multimodal Brain Image Analysis Laboratory, Centre for Brain Mapping & ADBS Neuroimaging Centre, National Institute of Mental Health and Neurosciences (NIMHANS), Bengaluru, Karnataka India; ^5^ Imaging Genetics Center, Mark and Mary Stevens Neuroimaging & Informatics Institute, University of Southern California, Marina del Rey, CA USA

## Abstract

**Background:**

We present the results of a task‐based fMRI study in early Alzheimer’s disease(mild cognitive impairment, MCI and mild Alzheimer’s disease, AD) using a novel‐fMRI memory paradigm suitable for use in patients with significant cognitive impairment having difficulties with remembering complex instructions.

**Method:**

The study samples comprised 65 patients with early AD(MCI n = 42; 21 males; mild AD n = 23; 16 males) and 26(14 males) elderly cognitively healthy control(eCHC) participants. The incidental encoding phase of the paradigm(7 minutes) comprised 110 trials of common objects(55 living and 55 non‐living trials which included 4 objects repeated 6 times each and 1 object repeated 5 times) while the intentional retrieval phase of the paradigm(7 minutes) comprised 55 trials of the 5 objects encoded during the previous phase(repeated 11 times each), and 55 new objects. Task‐based fMRI acquisitions were performed on a Philips Ingenia 3‐T MRI scannerand data was analysed using FEAT(FMRI Expert Analysis Tool) correcting for age, gender, education, and total brain volume.

**Result:**

Significantly increased activations spread across bilateral fronto‐parietal and occipital cortices were noted during incidental encoding of both ‘living’ and ‘non‐living’ stimuli in MCI when compared to eCHC and in mild AD when compared to MCI. However, reduced activations spread across bilateral central and parietal opercular cortices as well as frontal, temporal and parietal brain regions were noted during the intentional retrieval phase in response to both ‘memory’ and ‘non‐memory’ stimuli in MCI when compared to eCHC and in mild AD when compared to MCI.

**Conclusion:**

The increased brain activations of brain regions involved in during the incidental encoding phase reflects effortful encoding of novel stimuli in patients with early AD. The brain regions that showed reduced activations during the intentional retrieval phase have been previously reported to be involved in various processes that underlie retrieval of previously encoded memory traces. The results of the study support the utility of this novel‐fMRI memory paradigm as a tool suitable for brain imaging research in cognitively impaired research participants.